# Capturing patients’ needs in casemix: a systematic literature review on the value of adding functioning information in reimbursement systems

**DOI:** 10.1186/s12913-016-1277-x

**Published:** 2016-02-03

**Authors:** Maren Hopfe, Gerold Stucki, Ric Marshall, Conal D. Twomey, T. Bedirhan Üstün, Birgit Prodinger

**Affiliations:** 1 Swiss Paraplegic Research, 6207 Nottwil, Switzerland; 2 Department of Health Sciences & Health Policy, University of Lucerne, 6002 Lucerne, Switzerland; 3 National Centre for Classification in Health, Faculty of Health Sciences, University of Sydney, Lidcombe, NSW 2141 Australia; 4 Faculty of Social and Human Sciences, School of Psychology, University of Southampton, Southampton, SO17 1BJ UK; 5 World Health Organization, Classifications, Terminologies and Standards, 1211, Geneva, 27 Switzerland

**Keywords:** Functioning information, DRG, Casemix, Systematic review, Resource utilization

## Abstract

**Background:**

Contemporary casemix systems for health services need to ensure that payment rates adequately account for actual resource consumption based on patients’ needs for services. It has been argued that functioning information, as one important determinant of health service provision and resource use, should be taken into account when developing casemix systems. However, there has to date been little systematic collation of the evidence on the extent to which the addition of functioning information into existing casemix systems adds value to those systems with regard to the predictive power and resource variation explained by the groupings of these systems. Thus, the objective of this research was to examine the value of adding functioning information into casemix systems with respect to the prediction of resource use as measured by costs and length of stay.

**Methods:**

A systematic literature review was performed. Peer-reviewed studies, published before May 2014 were retrieved from CINAHL, EconLit, Embase, JSTOR, PubMed and Sociological Abstracts using keywords related to functioning (‘Functioning’, ‘Functional status’, ‘Function*, ‘ICF’, ‘International Classification of Functioning, Disability and Health’, ‘Activities of Daily Living’ or ‘ADL’) and casemix systems (‘Casemix’, ‘case mix’, ‘Diagnosis Related Groups’, ‘Function Related Groups’, ‘Resource Utilization Groups’ or ‘AN-SNAP’). In addition, a hand search of reference lists of included articles was conducted. Information about study aims, design, country, setting, methods, outcome variables, study results, and information regarding the authors’ discussion of results, study limitations and implications was extracted.

**Results:**

Ten included studies provided evidence demonstrating that adding functioning information into casemix systems improves predictive ability and fosters homogeneity in casemix groups with regard to costs and length of stay. Collection and integration of functioning information varied across studies. Results suggest that, in particular, DRG casemix systems can be improved in predicting resource use and capturing outcomes for frail elderly or severely functioning-impaired patients.

**Conclusion:**

Further exploration of the value of adding functioning information into casemix systems is one promising approach to improve casemix systems ability to adequately capture the differences in patient’s needs for services and to better predict resource use.

**Electronic supplementary material:**

The online version of this article (doi:10.1186/s12913-016-1277-x) contains supplementary material, which is available to authorized users.

## Background

Being able to respond in a balanced way to patients’ and populations’ needs and expectations is vital for a good health system performance [1]. Thus, health systems are expected to develop functions that improve populations’ health and account for fair financial contribution while securing responsiveness to patients’ health needs [2]. The most visible and fundamental function of health systems for ensuring responsive and appropriate care are health services. Health services aim to provide health interventions to patients and populations who need them, when and where needed, taking into account not only curing of diseases but also mitigating the impact of a health condition on a person’s life [3]. At the same time, provision and maintaining of needed services is constrained by scarce resources and cost containment measures. Thus, health systems are challenged in developing payment systems for health services that ensure responsiveness to patient’s needs and at the same time follow the principles of efficiency, fairness and appropriateness.

In practice, a variety of payment systems exist, for example, fee-for-service (FFS), capitation, per diem, salary or payments based on patient classification systems [4–7]. Such systems are widely referred to as casemix systems. Attention has been focused increasingly on casemix systems, with approximately 70 % of all OECD countries [8] and more than 25 low-and middle-income countries [9] having adopted some sort of casemix system for reimbursement purposes.

Developed in the 1970s by Robert B Fetter at Yale University for managing patients in acute hospitals, casemix systems are continuous learning systems which aim to improve transparency, efficiency and quality in health service provision [10]. They provide a standardized method for describing the product of health services provided (e.g. a specific set of outputs provided by a hospital to a patient) and their respective resource utilization while linking this information to costs. The most commonly known and widely used casemix system is the Diagnosis Related Groups casemix system (DRGs), a system that groups acute hospital inpatients primarily based on routinely collected patient variables, such as demographic, diagnostic and therapeutic characteristics [11]. Building upon the success of DRGs, several casemix systems have been (further) developed within, [12] and beyond, [13] the acute care sector since. The cornerstone of these systems remains the grouping of patients into classes that are homogeneous in their resource utilization and at the same time remain clinically coherent [14, 15]. In order to be successful in predicting health service utilization, casemix systems, regardless of their setting, need to capture and use the most important determinants for resource consumption in order to adequately describe a case in terms of resource utilization and to account for case complexity. Patients with the same diagnosis and treatment can differ widely in their need for services, including the scale of inputs required to achieve their goals, based on their ability to conduct daily life. How well a casemix system is able to predict resource use is often measured in terms of proportion of variation explained by casemix classes, such as DRGs [16].

In order to synthesize casemix-based payment rates with actual resource consumption based on patients’ needs for services, research has been conducted to examine the predictive power of various characteristics in addition to diagnostic patient characteristics and provided therapies [17–20]. It has been argued that diagnosis and therapeutic information alone do not adequately capture differences in case complexity of patients, which has led to increasing interest in incorporating severity of illness measures, risk factors, comorbidity data or patient characteristics such as age and weight into the systems [21–24]. However, criticism remains that current diagnosis based casemix systems, such as DRG-type systems, do not sufficiently explain legitimate differences in resource utilization, costs and length of stay in their casemix groupings [25].

Existing evidence shows that in various settings the functional status of patients is a predictor for mortality, discharge destination, readmission rate [26], length of stay [27, 28] and costs [29]. Functioning, understood as a multi-dimensional and interactive concept encompassing body structures, body functions as well as people’s capacity and actual performance to conduct activities of daily living and participate in their desired lifestyle, constitutes a fundamental component for understanding health and how it plays out in everyday life [30, 31]. Unlike severity of illness, which describes the magnitude of a health condition, functioning information covers the impact of a health condition on a patient’s life and therefore the potential value that corrective interventions may have for the patient. Consequently, it has been argued, that functioning information, as one important determinant of health service provision and resource use, needs to be taken into account when (further) developing casemix systems [32].

In addition, it has been shown, that functioning information complements diagnosis information and reflects patients’ need for services more adequately than focusing on medical aspects alone [33, 34]. Thus, functioning information constitutes a relevant component for determining patients’ health care need and hence respective resource use. This indicates that patient characteristics that go beyond the level of disease severity are promising candidates for better describing differences in patients’ needs and complexity. These characteristics may more adequately reflect resource utilization in casemix systems while at the same time make them more clinically meaningful descriptors of the cases.

To date, there exists little systematic evidence pertaining to whether the addition of functioning information into existing casemix systems adds value to those systems with regard to the predictive power and variation explained by the groupings of these systems. Therefore, the objective of this research was to examine existing evidence on the value of adding functioning information into casemix systems with respect to resource use as measured by costs and length of stay. A descriptive review was conducted to identify the current state of knowledge and to discuss its implications for future developments in financing health services that best meet patients’ needs.

## Method

### Study design

A systematic literature review was performed using standard literature databases to identify scientific publications that have examined the value of integrating functioning information into casemix systems. The process involved four steps: electronic literature search, article selection, data extraction and descriptive synthesis of results. The PRISMA guideline was applied to ensure transparent reporting of this systematic literature review [35]. The PRISMA checklist is provided as Additional file 1.

### Electronic literature search

The search was conducted in May 2014, without time limit, using the following databases covering medical, social and economic disciplines: Cumulative Index to Nursing and Allied Health Literature (CINAHL), American Economic Association’s electronic database (EconLit), Excerpta Medica Database (EMBASE), Journal Storage (JSTOR), PubMed and Sociological Abstracts.

A standardized search strategy for all databases was developed with only minor adaptations in order to account for database peculiarities. We combined search terms related to functioning (‘*Functioning*’, ‘*Functional status*’, ‘Function*’, ‘*ICF*’. ‘*International Classification of Functioning*, *Disability and Health*’, ‘*Activities of Daily Living*’ or ‘*ADL*’) with search terms related to casemix systems. The search terms for casemix systems include some commonly known and implemented casemix systems in acute and non-acute settings in order to account for the ongoing advancement and distribution of these systems across different sectors (‘*Casemix*’, ‘*case mix*’, ‘*Diagnosis Related Groups*’). Furthermore the search terms ‘*Function Related Groups*’, ‘*Resource Utilization Groups*’, or ‘*AN*-*SNAP*’ were used to specifically address casemix systems which explicitly use functioning information as key grouping variable. The search strategies are displayed in Additional file 2. In addition, a hand search of reference lists of included articles was conducted by reviewing the bibliographies of the included articles.

### Article screening and selection

The selection of articles was conducted in a step-wise process. In the first step, abstracts were screened and categorized based on pre-defined inclusion and exclusion criteria. Studies were included if they gave reference to functioning, functional status, ICF, International Classification of Functioning, Disability and Health, ADL, activities of daily living, or other domains of functioning (primarily Activity & Participation of ICF), such as dressing, self-care, self-reported health status; if they were either empirical studies on evaluation of casemix systems or approaches to grouping with comparative component (RCTs, pre-/post or before/after study design, cohort study), systematic reviews, meta analyses on casemix systems or approaches to grouping or empirical studies with comparison of people with different levels of functioning (or other terms as stated above) (e.g. studies that classified or stratified people by functional status or levels of ADL) in the context of casemix systems; and if they were published between 1977, development year of the first casemix system, and May 2014. Only studies that directly assessed functioning information were included. Studies using proxy indicators for functioning, such as discharge destination or referrals to therapists were excluded. Furthermore, studies, which were not directly related to resource utilization, as described by costs or length of stay, were excluded. In addition, studies had to be published in peer-reviewed international journals in English or German language, the languages available to the researchers,. Conceptual papers, expert opinion and theory development articles were excluded.

For reliability, the article selection (20 % of the abstract screening) was conducted in parallel by two independent researchers (MH and CT). In case of disagreement, the pros and cons for including the article were discussed with a third reviewer (BP) to come to a final decision. When the information in the abstract was not sufficient to assign the article, the article was included into the full text screening process.

### Data extraction

Information about study aims, design, country, setting, methods, outcome variables and key results was extracted. The operationalization of functioning variables was retrieved in detail. Information regarding the authors’ discussion of results, study limitations and implications of findings was extracted. Only relevant aspects of the study with regard to our research question were considered (e.g. only subsample provided information on functioning information). Data extraction was conducted by two researchers (MH and CT) independent from each other for more than 40 % of the included studies, solving disagreement by consensus. Data extraction and analyses were conducted and documented using Microsoft Excel 2010.

### Descriptive synthesis of results

For data synthesis, the characteristics of included studies were reviewed and recorded first. Then, key results on the value of adding functioning information into casemix systems as well as qualitative information regarding the implications of their findings were examined. The credibility of the studies was assessed using the Strengthening the Reporting of Observational Studies in Epidemiology (STROBE) guidelines for reporting observational studies [36, 37]. Given the lack of comparability of casemix systems and their dependency on the context of health system they are embedded in, we did not intend to synthesize results into a meta-analysis. Data extraction and presentation was conducted in an iterative process reviewing and interpreting extracted data against the full text of included studies to assure an accurate and consistent presentation of results.

## Results

### Literature search flow

**Fig. 1:**
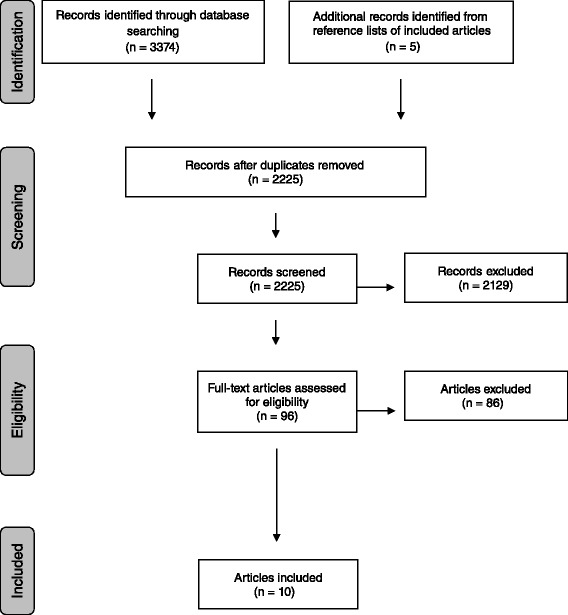
Flowchart of the systematic literature review

The electronic literature search resulted in 3379 identified records (Fig. 1). The reference search of included studies did not reveal additional articles for inclusion. After duplicates were removed, 2225 abstracts records were screened for eligibility with an agreement rate of 94 %. Based on the abstract screening, 2129 articles were excluded mainly because studies did not examine or evaluate casemix systems in the context of reimbursement but rather they used the terms relating to functioning and casemix as description of the population under study or as outcome parameters rather than as classification variables for comparing different groupings. In total, 96 articles qualified for more detailed screening at full-text level, of these 86 articles were excluded. Main reasons for exclusion in this screening phase included that the casemix systems concerned had been developed based on or included functioning information, the comparison of existing casemix systems already included functioning information. Therefore information on systems performance with and without functioning information could not be determined. In other excluded studies functioning was not assessed directly but inferred through proxies, such as referrals to therapists. In others, investigation of predictive power of functioning information was not related to casemix systems except within the introduction, discussion or conclusion.

In the final analysis 10 studies were included. The assessment of the included studies using the STROBE guidelines for reporting observational studies showed that in all studies the variables were described clearly. However, the detail of information on data sources varied across studies (see also Additional file 3).

### Study characteristics

The majority of the included articles were published between 2001 and 2010 (*n* = *7*). Five studies were conducted in North America, mostly particularly in the United States, four in Europe, and only one study was conducted in the Asian region (*Singapore*). Populations under study were mainly elderly patients with a mean age between 66 and 80 years (*n* = *3*) or older (*n* = *4*). The study characteristics, are summarized in Table 1.

**Table 1:** Study characteristics of included articles

			(*n* = *10*)
*A*) *Publication year*			
	a1	1991–2000	3
	a2	2001–2010	7
*B*) *Country*			
	b1	Asia	1
	b2	Europe	4
	b3	North America	5
*C*) *Study design*			
	c1	Prospective study	5
	c2	Retrospective study	4
	c3	Cross sectional study	1
*D*) *Mean*/*Median age* ^*a*^			
	d1	<65 yrs.	3
	d2	66–80 yrs.	3^b^
	d3	>80 yrs.	4

^*a*^If not indicated by the authors, means were estimated based on age distribution presented in the article

^b^One out of five DRGs had a mean age above 80 yrs

### Operationalization of functioning

Table 2 shows the various aspects of the operationalization of functioning extracted in the included studies. Four studies provide detailed information on how the functional variables were assessed. The remaining studies (*n* = *6*) only give summary information, such as “cognitive status” [38], “mobility” [39], “functional level before and after stroke” [40], “independent/dependent in 1 or more ADLs” [41], “self-care functions” [42], or “global assessment of functioning” [43] without any further variable specifications. In those studies that provided further specification, the most commonly assessed aspects of functioning were toileting (*n* = *3*), eating (*n* = *3*), bathing (*n* = *2*), dressing (*n* = *2*), and transferring (*n* = *2*) [44–47].

**Table 2:** Assessment of functioning in the studies (studies are listed in alphabetical order)

*Author*	*Specification of functioning variables*	*Mode of data collection*	*Time point of data collection*
Carpenter et al. (2007)	Indoor locomotion, eating, usage of toilets, personal hygiene, decision making, memory, making self-understood	Nurse assessment	Within 24 h of admission
Chuang et al. (2003)	Bathing, dressing, eating, toileting, transferring from a bed to a chair	Patient (or primary nurse/caregiver) interviews	On admission
Covinsky et al. (1997)	Bathing, dressing, grooming, transferring, eating, toileting	Interview of primary nurse or patient reports if nurse was not available	Within 48 h of admission
Dunstan et al. (1996)	Mobility	Expert assessment	Within 1^st^ week of admission
Evers et al. (2002)	Functional level before and after stroke	Maastricht Stroke Registry Hospital records	Not specified
Herwig et al. (2009)	Global assessment of functioning	Expert assessment using the GAF questionnaire	Not specified
Phillips & Hawes (1992)	Cognitive status	Dual expert assessments using orientation measures	24-h period
Pietz et al. (2004)	Physical functioning, role limitations resulting from physical problems, bodily pain, general health perceptions, energy/vitality, social functioning, role limitations resulting from emotional problems, mental health	Survey using the SF-36 questionnaire for Veterans	Within FY1998
Sahadevan et al. (2004)	Independent/dependent in 1 or more basic ADL	Not specified	On admission & at discharge
Warner et al. (2004)	Self-care functions	Veterans Affairs Spinal Cord Dysfunction Registry	Within the year 1995

*ADL* Activities of Daily Living, *FY* fiscal year (from October, 1^st^-September 30^th^), *GAF* Global Assessment of Functioning, *SF*-*12* 12-item Short Form Health Survey, *SF*-*36* 36-item Short Form Health Survey

The mode of data collection for functioning information ranged from direct patient or proxy interviews (*n* = *2*) to expert assessments (*n* = *4*), use of standardized questionnaires or surveys (*n* = *1*), as well as to information obtained from medical records or patient registries (*n* = *2*). One study did not specify the mode of data collection for functioning information [41]. The majority of the studies collected functioning information within a time period up to one week of admission (*n* = *6*). One of those studies collected functioning information on admission and at discharge [41]. The remaining studies (*n* = *4*) either provided a timeframe ranging by 24 h and one year (*n* = *2*) or did not specify the time point of data collection (*n* = *2*).

### The value of adding functioning information into casemix systems

Table 3 provides a summary of the evidence of the value of adding functioning information into casemix systems. The presentation of the results follow the principle of displaying a baseline model and subsequently the models which have been modified according to the respective study design and research question. These studies examine the increase in explained variance in outcome variables, the improvement in predictive power for outcome variables and the differences in outcome variables for patients with different levels of functioning in the context of casemix systems.

**Table 3:** Evidence for adding functioning information into casemix systems

*Author and year*	*Study characteristics* ^*a*^	*Setting & sample size* ^*b*^	*Type of casemix*	*Model*(*s*)	*Key results* ^c^
Costs					
Covinsky et al. (1997)	a1, b3, c1, d3	General medical service at a teaching hospital *n* = 823	DRG	*hospitalization costs*:	*hospitalization costs* (*measured in units*):
				Model 1*: Dependent in 0 ADL	*Model 1:* 100
				*Model 2:* Dependent in 1–3 ADLs	*Model 2:* 112 (99–126)
				*Model 3:* Dependent in 4–5 ADLs	*Model 3:* 142 (125–162)
				*Model 4:* Dependent in 6 ADLs	*Model 4:* 150 (131–172)
				* *all models controlled for Acute Physiology Score*, *Charlson score*, *age*, *race*, *gender*, *admission from nursing home and diagnosis related group cost weight*	
Evers et al. (2002)	a2, b2, c1, d2	Hospital *n* = 731	DRG	*Explained variance in costs directly related to medical care*:	*Explained variance costs directly related to medical care* (*R* ^*2*^):
				Total costs:	Total costs:
				*Model 1:* DRGs	*Model 1:* 0.338
				*Model 2:* DRGs + Need factors (includes functioning information among others)	*Model 2:* 0.547
				*Model 3:* DRGs + Need factors + Enabling factors + Predisposing factors + First order interactions (includes interactions between functioning and gender)	*Model 3: *0.611
				Diagnostic costs:	Diagnostic costs:
				*Model 1:* DRGs	*Model 1:* 0.168
				*Model 2:* DRGs + Need factors (includes functioning information among others)	*Model 2:* 0.362
				*Model 3:* DRGs + Need factors + Enabling factors + Predisposing factors + First order interaction (includes interactions between functioning and gender)	*Model 3: *0.407
				Therapeutic costs:	Therapeutic costs:
				*Model 1:* DRGs	*Model 1:* 0.377
				*Model 2:* DRGs + Need factors (includes functioning information among others)	*Model 2:* 0.483
				*Model 3:* DRGs + Need factors + Enabling factors + Predisposing factors + First order interactions (includes interactions between functioning and gender)	*Model 3:* 0.533
Chuang et al. (2003)	a2, b3, c2, d3	General medical service at a teaching hospital *n* = 1612	DRG	*Hospital costs*:	*Hospital costs* (*in* $):
				All patients:	All patients:
				*Model 1:* Independent in ADL on admission	*Model 1:* $4,060
				*Model 2:* Dependent in ADL on admission	*Model 2:* $5,300
				DRG weight <0.9:	DRG weight <0.9:
				*Model 1:* Independent in ADL on admission	*Model 1*: $3,090
				*Model 2:* Dependent in ADL in admission	*Model 2:* $4,130
				DRG weight 0.9-1.0:	DRG weight 0.9-1.0:
				*Model 1:* Independent in ADL on admission	*Model 1:* $3,560
				*Model 2:* Dependent in ADL on admission	*Model 2:* $4,440
				DRG weight 1.0-1.2:	DRG weight 1.0–1.2:
				*Model 1:* Independent in ADL on admission	*Model 1:* $3,940
				*Model 2:* Dependent in ADL on admission	*Model 2: *$4,840
				DRG weight >1.2:	DRG weight >1.2:
				*Model 1:* Independent in ADL on admission	*Model 1:* $6,560
				*Model 2:* Dependent in ADL on admission	*Model 2*: $8,250
				All patients adjusted for DRG weight:	All patients adjusted for DRG weight:
				*Model 1:* Independent in ADL on admission	*Model 1:* $4,140
				*Model 2:* Dependent in ADL on admission	*Model 2:* $5,240
				All patients adjusted for age, race, sex, Charlson Comorbidity score, APACHE II score, admission from nursing home and DRG weight:	All patients adjusted for age, race, sex, Charlson Comorbidity score, APACHE II score, admission from nursing home and DRG weight:
				*Model 1:* Independent in ADL on admission	*Model 1:* $4,220
				*Model 2:* Dependent in ADL on admission	*Model 2:* $5,200
Pietz et al. (2004)	a2, b3, c3, d1	VA medical centers primary care patients *n* = 35337	ACG-based ADGs	*Model’s ability to predict costs for FY 1998 and FY 1999*:	*Model*’*s ability to predict costs for FY 1998 and FY 1999* (*R* ^*2*^):
				Cost 1998:	Cost 1998:
				*Model 1:* ACGs	*Model 1:* 0.277
				*Model 2:* age, gender, ADGs, PCS, MCS,	*Model 2:* 0.294
				*Model 3:* age, gender, ADGs, 8 items	*Model 3:* 0.298
				Cost 1999:	Cost 1999:
				*Model 1: *ACGs	*Model 1: *0.070
				*Model 2:* age, gender, ADGs, PCS, MCS,	*Model 2:* 0.085
				*Model 3:* age, gender, ADGs, 8 items	*Model 3:* 0.087
				MAPE for costs 1999:	MAPE for 10th decile for costs 1999:
				*Model 1:* age, gender, ADGs	*Model 1:* $23440
				*Model 2:* age, gender, ADGs, 8 items	*Model 2:* $23204
Length of stay					
Dunstan et al. (1996)	a1, b2, c1, d3	Geriatric Medicine Service *n* = 400	development of new system (ACME)	*Explained variance for Length of Stay*:	*Explained variance for Length of Stay* (%):
				Model:	Model:
				*Model 1: *CMIX*	*Model 1:* 19.5 %
				*Model 2:* Presenting Illness (PI) + Functional Status (FX)	*Model 2: *19.2 %
				*Model 3:* PI	*Model 3: *13.0 %
				*Model 4:* FX	*Model 4:* 14.1 %
				Model + center:	Model + center:
				*Model 1:* CMIX*	*Model 1:* 25.2 %
				*Model 2:* PI + FX	*Model 2: *25.0 %
				*Model 3:* PI	*Model 3: *19.6 %
				*Model 4:* FX	*Model 4: *19.3 %
				Model + center + age + sex:	Model + center + age + sex:
				*Model 1:* CMIX*	*Model 1: *25.2 %
				*Model 2:* PI + FX	*Model 2:* 25.0 %
				*Model 3:* PI	*Model 3: *20.1 %
				*Model 4:* FX	*Model 4: *19.4 %
				*CMIX is a three-*level score calculated by simple addition of the 0 and 1 scores of PI and FX*.	
Sahadevan et al. (2004)	a2, b1, c1, d2	Acute care hospital Department of Geriatric Medicine & General Medicine Department *n = 232*	DRG	*Variance explained in actual Length of Stay*:	*Variance explained in actual Length of Stay* (*adjusted R* ^*2*^):
				*Analysis with outliers*:	*Analysis with outliers*:
				Length of stay (all subjects):	Length of stay (all subjects):
				*Model 1: *DRG’s trimmed ALOS	*Model 1:* 8 %
				*Model 2:* Functional status at discharge, total number of referrals to therapists, trimmed ALOS	*Model 2: *28 %
				Interdepartmental differences in Length of stay (subjects with common DRG):	Interdepartmental differences in Length of stay (subjects with common DRG):
				*Model 1:* Department factor + DRG’s trimmed ALOS	*Model 1:* 23 %
				*Model 2:* Functional profile at discharge, total number of referrals to therapists, trimmed ALOS, department factor	*Model 2: *31.4 %
				*Analysis without outliers*:	*Analysis without outliers*:
				Length of stay (all subjects):	Length of stay (all subjects):
				*Model 1:* DRG’s trimmed ALOS	*Model 1:* 23.8 %
				*Model 2:* Overall functional profile at admission, total number of therapy referrals, trimmed ALOS	*Model 2:* 31.4 %
				Interdepartmental differences in Length of stay (subjects with common DRG):	Interdepartmental differences in Length of stay (subjects with common DRG):
				*Model 1:* Department factor, DRG’s trimmed ALOS	*Model 1:* 28.1 %
				*Model 2:* Overall functional profile at admission, trimmed ALOS, referrals to medical social worker, department factor	*Model 2:* 34.5 %
Carpenter et al. (2007)	a2, b2, c1, d2	Hospital *n* = 1685	HRG (equivalent to DRG)	*Difference in actual Length of Stay & predicted Length of Stay*:	*Difference in actual Length of Stay & predicted Length of Stay* (*Ratio & 95* % *CI*)
				All patients:	All patients:
				*Model 1:* low and medium ADL score	*Model 1:* 1
				*Model 2:* high ADL score	*Model 2: *1.40 (1.26–1.56)
				Stroke:	Stroke:
				*Model 1:* low and medium ADL score	*Model 1: *1
				*Model 2:* high ADL score	*Model 2:* 1.67 (1.23–2.26)
				Acute respiratory infection:	Acute respiratory infection:
				a)	a)
				*Model 1:* medium ADL score	*Model 1: *1
				*Model 2:* high ADL score	*Model 2:* 1.44 (1.16–1.80)
				b)	b)
				*Model 1: *low ADL score	*Model 1:* 1
				*Model 2:* medium ADL score	*Model 2:* 1.37 (1.01–1.85)
				Chronic obstructive pulmonary disease:	Chronic obstructive pulmonary disease:
				*Model 1: *low and medium ADL score	*Model 1:* 1
				*Model 2:* high ADL score	*Model 2:* 1.21 (1.04–1.53)
				Falls:	Falls:
				*Model 1:* low and medium ADL score	*Model 1: *1
				*Model 2: *high ADL score	*Model 2:* 1.68 (1.23–2.28)
				* *all models controlled for healthcare resource group length of stay*, *hospital*, *discharge destination*, *admission source and age*	
Herwig et al. (2009)	a2, b2, c2, d1	University hospital, Psychiatry *n *= 613	development of new system based on AMDP	*Predicted variation in Length of Stay*:	*Predicted variation Length of Stay* (%):
				*Model 1:* AMDP Syndromes (Psychopathological Syndromes)*	*Model 1:* 5,9 %
				*Model 2:* AMDP Syndromes + Age at admission + Global assessment of functioning + clinical global impressions + voluntary admission + own apartment**	*Model 2:* 19,8 %
				*n = 998	
				**n = 613	
Warner et al. (2004)	a2, b3, c2, d1	Inpatient & Outpatient Veterans *n *= 5888	ACG & DCG	*Predicting inpatient*, *outpatient and total days of care*:	*Predicting inpatient*, *outpatient and total days of care* (*R* ^*2*^):
				DCG:	DCG*:
				*Model 1:* Age/sex + HCCs	*Model 1:* Inpatient days of care (IP): 0.36; Outpatient days of care (OP): 0.33; Both: 0.30
				*Model 2:* Functionally enhanced*	*Model 2: *IP: 0.36; OP: 0.33; Both: 0.30
				ACG:	ACG*:
				*Model 1: *Age/sex + ADGs	*Model 1:* IP: 0.15; OP: 0.28; Both: 0.20
				*Model 2:* Functionally enhanced*	*Model 2:* IP: 0.19; OP: 0.28; Both: 0.22
				* *Functionally enhanced*: *ACG*/*DCG* + *age*, *gender* + *self*-*reported functional measure*	* *n* = *2347 for inpatient days of care and n* = *5888 for outpatient days of care*
Resource provision					
Phillips & Hawes (1992)	a1, b3, c2, d3	Nursing care units *n* = 1792	RUG-II	*Explained variation in resource provision by time*:	*Explained variation in resource provision by time* (*R* ^*2*^):
				Licensed time:	Licensed time:
				*Model 1:* RUG-II	*Model 1:* 0.14
				*Model 2: *RUG-II with cognitive variables	*Model 2:* 0.16
				Aide time:	Aide time:
				*Model 1:* RUG-II	*Model 1:* 0.39
				*Model 2:* RUG-II with cognitive variables	*Model 2:* 0.39
				Total time:	Total time:
				*Model 1:* RUG-II	*Model 1:* 0.40
				*Model 2: *RUG-II with cognitive variables	*Model 2:* 0.40

*ACG* Adjusted Clinical Groups, *ACME* Admission Case-Mix System for the Elderly, *ADG* Adjusted Diagnostic Groups, *ADL* activities of daily living, *ALOS* average length of stay, *AMDP* Arbeitsgemeinschaft für Methodik und Dokumentation, *CI* confidence interval, *DCG* Diagnostic Costing Groups, *DRG* Diagnosis Related Groups, *FX* functional status, *HCC* Hierarchical Condition Categories, *HMO* health maintenance organization, *HRG* Healthcare Resource Groups, *IP* inpatient days of care, *MAPE* Mean Absolute Predicted Error, *MCS* Mental Component Score, *ns* not specified, *OP* outpatients days of care, *PCS* Physical Component Score, *PI* presenting illness, *RUG*-*II* Resource Utilization Groups Version II, *VA* veteran affairs

^a^See Table 1 study characteristics

^b^Information in the table is presented as stated by author

^c^Presentation of figures of key results for each model are aligned with the presentation of results by authors of the study

### Costs

Four studies address the value of adding functioning information into casemix systems with costs as the outcome parameter. Apart from one exception, the casemix system under investigation in these studies is the DRG casemix system in hospital settings. Some of the studies provide evidence that in older patients higher dependency in ADL is significantly associated with higher costs of hospitalization even after adjusting for DRG cost weights and other patient characteristics. Patients with a higher dependency in ADLs cost up to 50 % more than patients independent in ADLs [44]. Another study shows that including functioning information into a DRG casemix system in the hospital setting increases the variance explained in costs directly related to medical care from 34 % up to 61 % [40]. There is also a modest improvement with regard to the predictive power of costs in the Johns Hopkins ACG casemix system for primary care patients [46].

### Length of stay

Five studies investigate the effects of adding functioning information to casemix systems with regard to the outcome parameter length of stay (LOS). One study used inpatient/outpatient and total days of care as the outcome variable which follows the underlying principle of LOS. This study was therefore included in this section. LOS was used in several studies as a proxy for resource utilization. The studies provide evidence that adding functioning information into DRG casemix systems in the acute hospital setting increases the explained variance in actual LOS of elderly patients from 8 % up to 28 %. Even when excluding outliers from the analyses, the amount of variance explained increase from 23.8 % up to 31.4 % [41]. In another study, the difference between predicted LOS and actual LOS was examined in the Healthcare Resource Groups (HRGs) casemix system. HRGs are analogous to DRGs. Consistently with the other studies, patients dependent in physical functioning stayed up to 40 % longer than patients with lower dependency when controlling for HRG [47]. For systems being developed for other settings, such as geriatric medicine or mental health, the addition of functioning information increased explained variance for LOS from 13.0 % to 19.2 % or from 5.9 % to 19.8 % [39, 43]. Adding functioning information into Johns Hopkins ACG/DCG casemix systems for predicting inpatient/outpatient and total days of care for inpatient and outpatient veterans did not reveal great improvement in the systems’ predictive power in the study extracted [42].

### Resource provision

One study examined the addition of cognitive functioning into a casemix system that already accounts for different levels of patient’s functioning. As might be expected, the results did not reveal any improvement in explained variation in resource use for nursing home patients [38].

### Synthesis of qualitative information regarding implications of findings

In addition to the empirical evidence generated by the studies, most authors also highlight the value of adding functioning information into casemix systems from a conceptual perspective. The authors acknowledge in their discussions that current casemix systems do not adequately capture and describe the differences in patients’ needs for services to adequately predict health service resource utilization. Authors argue that especially DRG casemix systems discriminate against hospitals treating frail elderly or patients with severely limited levels of functioning. While three studies do not highlight or discuss the value of functioning information, they do in some cases question the precision of the measurement of functioning they used [46] and critically discuss the overall weak performance of the casemix system under study [43]. In other cases they discuss the existence of relative merits in a casemix system that already accounts for certain aspects of functioning within their grouping process including where additional aspects of functioning did not reveal improvement [38].

## Discussion

This review provides evidence demonstrating that, in the context of improving casemix systems, functioning information is becoming recognized as an important factor for determining patients’ health care needs and respective resource use. The 10 included studies found to have shed light on this question, varied with regard to study design, setting of the care provided, casemix system under study, input variables and outcome variables. The detailed analysis of the studies shows that adding functioning information into casemix systems, where it is not already included, strengthens the predictive power of these systems and increases the variance explained with regard to cost or proxies for costs, such as LOS. The results suggest that DRG casemix systems can be improved in their accounting for indicators for resource use by adding functioning variables particularly in frail elderly or severely functioning-impaired patients.

These findings serve as first evidence for further studying the implications of functioning information for improving financing of health services to better meet patients’ needs. This relates to the technical improvement of predictive ability of systems and homogeneity in casemix groups, the information standards and requirements for integrating data into casemix systems and how improvement of casemix systems might change incentive mechanisms within health systems.

### Technical improvement of predictive ability of systems and homogeneity in casemix groups

Predictive ability and homogeneity in casemix groups are fundamental design components when looking at the success of casemix systems to predict resource utilization in terms of costs or proxies for costs, such as LOS. The results show that both, predictive ability and explained variance in casemix groups can be strengthened by adding functioning information into the systems. It is important to note that the amount of improvement varies with regard to setting, casemix system and outcome parameter. For example, adding functioning information into casemix systems, such as ACG/DCG casemix systems to predict costs or days of care in outpatient and primary care settings revealed modest to no improvement. One explanation could be that in these settings, other variables, such as social environment, availability and historic growth of health services as well as educational level might impact overwhelmingly the prediction of health service utilization. Improvements are more pronounced when adding functioning information into DRG casemix systems for elderly patients in hospital inpatient care settings. In this setting, the functional status of patients might play an important role to explain the high cost or length of stay outliers, especially for patients that are severely functioning impaired or where the acute care of a health condition is followed by rehabilitation care. From a technical point of view, casemix systems performance have been mainly evaluated in terms of predictive validity and resource homogeneity of case groups [48]. At the same time, casemix groupings need to remain clinical meaningful in order to ensure utility of the systems for clinical context. Increasing the number of groups based on more distinct patient characteristics, such as functional level of patients is one way to increase variance explained by the respective model [49]. Yet, the number of case groups should not exceed a reasonable and manageable limit. Furthermore, increasing predictive ability solely based on adding more variables might risk losing clinical meaningfulness of groupings [50]. Thus, discussions on how much improvement in predictive power is a criterion for better casemix systems’ performance needs to consider the trade-off between statistical predictive ability and clinical meaningfulness. The evidence from this review suggests that it will be worthwhile to further explore the value of adding functioning information both empirically and conceptually.

### Information standards and requirements for integrating functioning into casemix systems

Concerning the operationalization of functioning, the results show that functioning information that can be used covers a wide spectrum. Although some functioning variables were assessed more often than others, the variety of variables ranged from those often referred to activities of daily living, such as bathing, dressing and transferring to more complex constructs like bodily pain, cognitive status or restriction in socializing. A fundamental component for successful casemix systems to predict health service resource utilization is the accurate and precise coding of information that can be used in the grouping process of casemix classes. Thus, a clear conceptualization and consistent operationalization of functioning assessment and reporting is needed for this concept to become systematically included and coded in casemix systems. In addition, based on this review no conclusions can be drawn at this stage on the optimal type of assessment mode for functioning (e.g. patient interviews vs. expert assessments, dependency vs. difficulty). Studies varied widely with regard to mode of data collection and this may reflect the variety of instruments used across differing clinical areas. Although the majority of the included studies collected functioning information within a time period up to one week of admission, no conclusive statement can be made at this stage regarding the optimal time point of data collection. The results are only indicative and further research is required to standardize the methods for and timing of assessing functioning information in the context of casemix systems [51, 52]. While this is not a problem for individual studies itself, it impedes comparability across systems as well as preventing useful meta-analysis of the existing studies. At the same time, it is important to consider, that the cost of obtaining the information should not exceed the value of including the information in the system. That functioning information complements disease information is in line with movements towards the joint use of disease information and functioning information led by the WHO and their approach to incorporate aspects of functioning in the eleventh revision of International Classification of Diseases (ICD-11) [53–55]. Further exploring the value of the joint use of international standards for coding and reporting disease and functioning information in the context of casemix systems could be a promising way forward.

### Improvement of casemix systems as incentive mechanisms within health systems

Casemix systems are subject to the specific health system they are embedded in and they can constitute powerful incentive mechanisms within the health system [56]. Results of this review suggest that especially DRG casemix systems in hospital settings can be improved in adequately capturing resource use in terms of costs or LOS, particularly for frail elderly or patients with a more severe level of functioning impairment. These patients constitute a heterogeneous group with often complex medical, functional and psychological problems. Treatment does not only comprise curing of acute underlying disease but also taking care of patients’ complex functioning needs. The presented results raise concerns that hospitals treating those patients under the DRG casemix system run the risk of being incentivized to avoid or underservice highly resource intensive patients. This review provides valuable information for stakeholders who are challenged in developing reimbursement strategies for health services that ensure being responsive to changing patients’ and populations’ needs. Based on current trends, the percentage of the population over 65 years and older will increase to 26 % in 2050 (16 % in 2010) in the developed world and to 15 % (6 % in 2010) in the developing world [57]. Simultaneously chronic conditions–chiefly cardiovascular diseases, cancer, chronic respiratory diseases and diabetes–are predicted to account for 73 % of deaths and 60 % of diseases burden in the year 2020 (as compared to 60 % deaths and 43 % burden of diseases in 1999) [58]. It has been shown that DRG systems are struggling to explain the variation in costs and length of stay for those patients experiencing chronic conditions, such as stroke [59], acute myocardial infarction [60] or breast cancer [61]. The authors of these studies agree that considering additional patient-related variables can improve the overall performance of these systems, and this review indicates that functioning information might be one of these additional variables with the potential to improve the performance of DRG systems.

In summary, casemix systems need to be adapted to capture those factors that can better account for differential resource utilization particularly in elderly [62] and functioning-impaired patients in order to ensure delivery of health services that are responsive to patients’ needs and to avoid perverse incentives.

Health systems are highly complex with multiple stakeholders and competing interests. Casemix systems, as important tools for resource distribution within health systems, are subject to various influences and vested interests that go beyond predictive ability and homogeneity in case groups. Thus, when adapting and changing casemix systems, it is important to account for different arguments and to actively involve stakeholders from the beginning in the process. Learning from the experiences of countries which have attempted integrating functioning information can be a beneficial approach when fostering the discussion about adapting current casemix systems. This might not only include identifying the arguments from past experiences for or against including functioning information but also examining the current opinion on the potential value and opportunities for functioning information in casemix systems given the experiences stakeholders have now.

## Limitation

This review is subject to several limitations: First, the research question is limited to studies examining the effects of adding functioning information into casemix systems currently without such information. It excludes a broad range of studies dealing with systems that have been developed on or already include functioning information as one of the components of their systems. Second, the search only included peer-reviewed studies. The development and design of casemix systems involves many stakeholders and more detailed information on the value and reasoning of adding functioning information might be given in various policy development documents and the “grey literature”. Third, the credibility of selected studies was examined based on the STROBE guidelines for reporting observational studies. However, they are not intended to assess the methodological quality of the study per se but indicate some weaknesses of the reviewed studies. The level of detailed information reported varied across studies. In addition, it is important to note that while casemix systems around the world do have fundamental similarities with regard to design components and mechanisms, there are differences with regard to national adaptations in the grouping process and applications. Thus, the results have to be considered in the context of the specific health systems concerned.

## Conclusion

This review provides promising evidence suggesting that further exploring the value of adding functioning information into casemix systems represents one promising approach to improve these systems’ ability to adequately capture the differences in patient’s needs for services and to their ability to predict resource use in terms of costs particularly for frail elderly or severely functioning-impaired patients. However, there is no common agreement on how much improvement in predictive power is needed for “better” casemix systems performance. Moreover, discussions around improving financing of health services that meet patients’ needs should consider the trade-off between predictive ability and clinical meaningfulness of casemix groups. Building upon a common framework for operationalizing functioning information based on international standards could be a valuable option to proceed. This would require ensuring that such operationalization of functioning is fit for purpose in specific casemix systems for example by giving greater focus to outcomes for patients.

## Additional files

Additional file 1: **The PRISMA checklist.** (PDF 58 kb)

Additional file 2: **The search strategies.** (PDF 21 kb)

Additional file 3: **STROBE.** (PDF 28 kb)

## Abbreviations

ACG: Adjusted Clinical Groups
ADL: Activities of Daily Living
AN-SNAP: The Australian National Subacute and Non-Acute Patient Classification
DCG: Diagnostic Costing Groups
DRG: Diagnosis Related Groups
FFS: Fee-For-Service
HRG: Healthcare Resource Groups
ICD: International Classification of Diseases
ICF: International Classification of Functioning, Disability and Health
LOS: Length of Stay
OECD: Organisation for Economic Co-operation and Development
RCT: Randomized Controlled Trial
WHO: World Health Organization
