# Supplementary material for: Capturing patients’ needs in casemix: a systematic literature review on the value of adding functioning information in reimbursement systems
Source: BMC Health Serv Res. 2016 Feb 3;16:40. doi: 10.1186/s12913-016-1277-x (PMC4741002; doi:10.1186/s12913-016-1277-x)
Supplement: Supplementary file 2 — The search strategies. (PDF 21 kb) [file 12913_2016_1277_MOESM2_ESM.pdf]

Additional file 2: search strategy

PubMed (05.05.2014)

(Functioning[All Fields] OR "Functional status"[All Fields] OR (function[All Fields] OR function'[All Fields] OR function''[All Fields] OR function'alis[All Fields] OR function'in[All Fields] OR function's[All Fields] OR function1[All Fields] OR function2[All Fields] OR function28[All Fields] OR function2a[All Fields] OR function2gene[All Fields] OR function490[All Fields] OR function647[All Fields] OR functiona[All Fields] OR functionaal[All Fields] OR functionability[All Fields] OR functionable[All Fields] OR functionaction[All Fields] OR functionae[All Fields] OR functionai[All Fields] OR functionaing[All Fields] OR functionaires[All Fields] OR functionais[All Fields] OR functionaities[All Fields] OR functionakimageanalysis[All Fields] OR functional[All Fields] OR functional'[All Fields] OR functional''[All Fields] OR functional'noe[All Fields] OR functional'proximal[All Fields] OR functional's[All Fields] OR functionala[All Fields] OR functionalactivity[All Fields] OR functionalal[All Fields] OR functionalanalysis[All Fields] OR functionalanalytical[All Fields] OR functionaland[All Fields] OR functionalbeta[All Fields] OR functionalbiological[All Fields] OR functionalclass[All Fields] OR functionalconstipation[All Fields] OR functionaldiagnostic[All Fields] OR functionale[All Fields] OR functionalenzyme[All Fields] OR functionales[All Fields] OR functionalflow[All Fields] OR functionalgenomics[All Fields] OR functionalglycomics[All Fields] OR functionalgrazers[All Fields] OR functionalgroup[All Fields] OR functionalgroups[All Fields] OR functionali[All Fields] OR functionalied[All Fields] OR functionalimaging[All Fields] OR functionalis[All Fields] OR functionalisable[All Fields] OR functionalisation[All Fields] OR functionalisations[All Fields] OR functionalisative[All Fields] OR functionalise[All Fields] OR functionalised[All Fields] OR functionalised'[All Fields] OR functionalises[All Fields] OR functionalising[All Fields] OR functionalism[All Fields] OR functionalismo[All Fields] OR functionalisms[All Fields] OR functionalist[All Fields] OR functionalist'[All Fields] OR functionalist's[All Fields] OR functionalistic[All Fields] OR functionalistion[All Fields] OR functionalists[All Fields] OR functionalitate[All Fields] OR functionalitatea[All Fields] OR functionalitatii[All Fields] OR functionalitation[All Fields] OR functionalites[All Fields] OR functionalities[All Fields] OR functionalities'[All Fields] OR functionalitites[All Fields] OR functionality[All Fields] OR functionality'[All Fields] OR functionality's[All Fields] OR functionalitymay[All Fields] OR functionalizability[All Fields] OR functionalizable[All Fields] OR functionalizaed[All Fields] OR functionalizarea[All Fields] OR functionalizated[All Fields] OR functionalizating[All Fields] OR functionalization[All Fields] OR functionalization'[All Fields] OR functionalization5[All Fields] OR functionalizationed[All Fields] OR functionalizationof[All Fields] OR functionalizations[All Fields] OR functionalizatlon[All Fields] OR functionalizaton[All Fields] OR functionalizd[All Fields] OR functionalize[All Fields] OR functionalizeable[All Fields] OR functionalized[All Fields] OR functionalizers[All Fields] OR functionalizes[All Fields] OR functionaliziation[All Fields] OR functionalizied[All Fields] OR functionalizing[All Fields] OR functionalizion[All Fields] OR functionaliztion[All Fields] OR functionallimitations[All Fields] OR functionallized[All Fields] OR functionallly[All Fields] OR functionally[All Fields] OR functionally'[All Fields] OR functionallydependent[All Fields] OR functionallyidentical[All Fields] OR functionallywise[All

Fields] OR functionalmedicine[All Fields] OR functionalmitral[All Fields] OR functionalmolecular[All Fields] OR functionalmorphology[All Fields] OR functionalmri[All Fields] OR functionalnature[All Fields] OR functionalnet[All Fields] OR functionalneurons[All Fields] OR functionalnoda[All Fields] OR functionaloutcomes[All Fields] OR functionalpolarities[All Fields] OR functionalproteins[All Fields] OR functionalroles[All Fields] OR functionals[All Fields] OR functionals'[All Fields] OR functionalsimilarity[All Fields] OR functionalsolutions[All Fields] OR functionalstructure[All Fields] OR functionalstudies[All Fields] OR functionalties[All Fields] OR functionalvoice[All Fields] OR functionaly[All Fields] OR functionalysed[All Fields] OR functionalyzed[All Fields] OR functionalzation[All Fields] OR functionalziation[All Fields] OR functionamento[All Fields] OR functionamiento[All Fields] OR functionanalyzer[All Fields] OR functionand[All Fields] OR functionante[All Fields] OR functionantes[All Fields] OR functionare[All Fields] OR functionarea[All Fields] OR functionaresse[All Fields] OR functionaries[All Fields] OR functionaries'[All Fields] OR functionarii[All Fields] OR functionarios[All Fields] OR functionaris[All Fields] OR functionarissen[All Fields] OR functionarization[All Fields] OR functionary[All Fields] OR functionas[All Fields] OR functionassociated[All Fields] OR functionate[All Fields] OR functionates[All Fields] OR functionating[All Fields] OR functionation[All Fields] OR functionator[All Fields] OR functionbody[All Fields] OR functionc[All Fields] OR functioncdots[All Fields] OR functiond[All Fields] OR functione[All Fields] OR functioneal[All Fields] OR functioned[All Fields] OR functioneel[All Fields] OR functioneelanatomisch[All Fields] OR functioneert[All Fields] OR functionel[All Fields] OR functionele[All Fields] OR functionelisa[All Fields] OR functionelle[All Fields] OR functionellen[All Fields] OR functioneller[All Fields] OR functionelles[All Fields] OR functionellipsis[All Fields] OR functionellipsis'[All Fields] OR functionem[All Fields] OR functionen[All Fields] OR functioner[All Fields] OR functioneren[All Fields] OR functionerend[All Fields] OR functionerende[All Fields] OR functiones[All Fields] OR functionfunctions[All Fields] OR functiong[All Fields] OR functionh[All Fields] OR functionialized[All Fields] OR functionialy[All Fields] OR functionig[All Fields] OR functionilized[All Fields] OR functionimg[All Fields] OR functionin[All Fields] OR functioning[All Fields] OR functioning'[All Fields] OR functioning's[All Fields] OR functioninga[All Fields] OR functioningand[All Fields] OR functioningin[All Fields] OR functionings[All Fields] OR functionings'[All Fields] OR functioningt[All Fields] OR functioningthe[All Fields] OR functionioning[All Fields] OR functionisable[All Fields] OR functionization[All Fields] OR functionized[All Fields] OR functionl[All Fields] OR functionla[All Fields] OR functionlaized[All Fields] OR functionless[All Fields] OR functionless'[All Fields] OR functionlessness[All Fields] OR functionlike[All Fields] OR functionlization[All Fields] OR functionlized[All Fields] OR functionly[All Fields] OR functionment[All Fields] OR functionn[All Fields] OR functionnal[All Fields] OR functionnale[All Fields] OR functionnalization[All Fields] OR functionnalized[All Fields] OR functionnally[All Fields] OR functionnaly[All Fields] OR functionned[All Fields] OR functionnel[All Fields] OR functionnele[All Fields] OR functionnelle[All Fields] OR functionnelles[All Fields] OR functionnels[All Fields] OR functionnement[All Fields] OR functionnes[All Fields] OR functionning[All Fields] OR functiono[All Fields] OR functionof[All Fields] OR functionograph[All Fields] OR functionography[All Fields] OR functionome[All Fields] OR functionomes[All Fields] OR functionometric[All Fields] OR functionomic[All Fields] OR functionomics[All Fields] OR functionomics'[All Fields] OR functionpiso[All Fields] OR

functionplasma[All Fields] OR functionplaster[All Fields] OR functionpost[All Fields] OR functionprediction[All Fields] OR functionpsi[All Fields] OR functionrelationship[All Fields] OR functions[All Fields] OR functions'[All Fields] OR functions's[All Fields] OR functions1[All Fields] OR functionsal[All Fields] OR functionsand[All Fields] OR functionsaving[All Fields] OR functionse[All Fields] OR functionsf[All Fields] OR functionship[All Fields] OR functionsmduring[All Fields] OR functionsmeasured[All Fields] OR functionsrelated[All Fields] OR functionsreproduce[All Fields] OR functionsstorungen[All Fields] OR functionstate[All Fields] OR functionsurg[All Fields] OR functionswechsels[All Fields] OR functionswere[All Fields] OR functionswithin[All Fields] OR functiont[All Fields] OR functiontesting[All Fields] OR functionthere[All Fields] OR functiontional[All Fields] OR functiontrpc[All Fields] OR functionum[All Fields] OR functionwere[All Fields] OR functionwise[All Fields] OR functionx[All Fields] OR functiony[All Fields]) OR ICF[All Fields] OR "International Classification of Functioning, Disability and Health"[All Fields] OR "Activities of Daily Living"[All Fields] OR ADL[All Fields]) AND (Casemix[All Fields] OR "case mix"[All Fields] OR "Diagnosis Related Groups"[All Fields] OR "Function Related Groups"[All Fields] OR "Resource Utilization Groups"[All Fields] OR "AN-SNAP"[All Fields])

CINAHL (05.05.2014)

(Functioning OR “Functional status” OR Function* OR ICF OR “International Classification of Functioning, Disability and Health” OR “Activities of Daily Living” OR ADL) AND (Casemix OR “case mix” OR “Diagnosis Related Groups” OR “Function Related Groups” OR “Resource Utilization Groups” OR “AN-SNAP”)

EMBASE (05.05.2014)

#1	(Functioning or “functional status” or Function* or ICF or International Classification of Functioning, Disability and Health” or “Activities of Daily Living” or ADL).af.
#2	(Casemix or “case mix” or “Diagnosis Related Groups” or “Function Related Groups” or “Resource Utilization Groups” or “AN-SNAP”).af.
#3	#1 AND #2

Sociological Abstracts (05.05.2014)

(Functioning OR “Functional status” OR Function* OR ICF OR “International Classification of Functioning, Disability and Health” OR “Activities of Daily Living” OR ADL) AND (Casemix OR “case mix” OR “Diagnosis Related Groups” OR “Function Related Groups” OR “Resource Utilization Groups” OR “AN-SNAP”)

EconLit (05.05.2014)

#1 (Functioning or “functional status” or Function* or ICF or International Classification of Functioning, Disability and Health” or “Activities of Daily Living” or ADL).af.

#2 (Casemix or “case mix” or “Diagnosis Related Groups” or “Function Related Groups” or “Resource Utilization Groups” or “AN-SNAP”).af.

#3 #1 AND #2

EconLit (06.05.2014)

#1 (Functioning OR “Functional status” OR ICF OR “International Classification of Functioning, Disability and Health” OR “Activities of Daily Living” OR ADL) AND (Casemix OR “case mix” OR “Diagnosis Related Groups” OR “Function Related Groups”)

#2 (Functioning OR “Functional status” OR ICF OR “International Classification of Functioning, Disability and Health” OR “Activities of Daily Living” OR ADL) AND ("Resource Utilization Groups” OR "AN-SNAP")
