# Supplementary material for: Capturing patients’ needs in casemix: a systematic literature review on the value of adding functioning information in reimbursement systems
Source: BMC Health Serv Res. 2016 Feb 3;16:40. doi: 10.1186/s12913-016-1277-x (PMC4741002; doi:10.1186/s12913-016-1277-x)
Supplement: Supplementary file 3 — STROBE. (PDF 28 kb) [file 12913_2016_1277_MOESM3_ESM.pdf]

	Item No	Recommendation	Carpenter et al. (2007) Reported	Chuang et al. (2003) Reported	Covinsky et al. (1997) Reported	Dunstan et al. (1996) Reported	Evers et al. (2002) Reported	Herwig et al. (2009) Reported	Philips & Hawes (1992) Reported	Pietz et al. (2004) Reported	Sahadevan et al. (2004) Reported	Warner et al. (2004) Reported
**Title and abstract**	1	(*a*) Indicate the study's design with a commonly used term in the title or the abstract	yes	yes	no	no	no	no	no	yes	no	no
		(*b*) Provide in the abstract an informative and balanced summary of what was done and what was found	yes	yes	yes	yes	yes	no	no	yes	yes	yes
**Introduction**												
Background/rationale	2	Explain the scientific background and rationale for the investigation being reported	yes	yes	yes	yes	yes	yes	yes	yes	yes	yes
Objectives	3	State specific objectives, including any prespecified hypotheses	yes	yes	yes	yes	yes	yes	yes	yes	yes	yes
**Methods**												
Study design	4	Present key elements of study design early in the paper	yes	yes	yes	yes	yes	yes	yes	yes	yes	yes
Setting	5	Describe the setting, locations, and relevant dates, including periods of recruitment, exposure, follow-up, and data collection	yes	yes	yes	no	yes	yes	yes	yes	yes	yes
Participants	6	(*a*) *Cohort study* —Give the eligibility criteria, and the sources and methods of selection of participants. Describe methods of follow-up	yes	yes	yes	yes	no	yes		yes	yes	
		Case-control study —Give the eligibility criteria, and the sources and methods of case ascertainment and control selection. Give the rationale for the choice of cases and controls										
		Cross-sectional study —Give the eligibility criteria, and the sources and methods of selection of participants							no			yes

		(*b*) *Cohort study*—For matched studies, give matching criteria and number of exposed and unexposed<br>*Case-control study*—For matched studies, give matching criteria and the number of controls per case	n.a.	n.a.	n.a.	n.a.	n.a.	n.a.		n.a.	n.a.	
Variables	7	Clearly define all outcomes, exposures, predictors, potential confounders, and effect modifiers. Give diagnostic criteria, if applicable	yes	yes	yes	yes	yes	yes	yes	yes	yes	yes
Data sources/ measurement	8*	For each variable of interest, give sources of data and details of methods of assessment (measurement). Describe comparability of assessment methods if there is more than one group	yes	yes	yes	yes	no	no	yes	yes	no	yes
Bias	9	Describe any efforts to address potential sources of bias	yes	no	yes	yes	yes	yes	yes	yes	yes	yes
Study size	10	Explain how the study size was arrived at	yes	yes	yes	yes	yes	yes	yes	yes	yes	yes
Quantitative variables	11	Explain how quantitative variables were handled in the analyses. If applicable, describe which groupings were chosen and why	yes	yes	yes	yes	yes	no	no	yes	yes	yes
Statistical methods	12	(*a*) Describe all statistical methods, including those used to control for confounding	yes	yes	yes	yes	yes	yes	no	yes	yes	yes
		(*b*) Describe any methods used to examine subgroups and interactions	yes	yes	n.a.	yes	n.a.	n.a.	n.a.	yes	yes	yes
		(*c*) Explain how missing data were addressed	no	yes	yes	no	yes	yes	yes	yes	n.a.	yes
		(*d*) *Cohort study*—If applicable, explain how loss to follow-up was addressed<br>*Case-control study*—If applicable, explain how matching of cases and controls was addressed	n.a.	n.a.	yes	n.a.	n.a.	n.a.		n.a.	n.a.	

		Cross-sectional study —If applicable, describe analytical methods taking account of sampling strategy							n.a.			n.a.
		(*e*) Describe any sensitivity analyses	no	yes	no	no	no	no	no	no	no	no
**Results**												
Participants	13*	(a) Report numbers of individuals at each stage of study—eg numbers potentially eligible, examined for eligibility, confirmed eligible, included in the study, completing follow-up, and analysed	yes	yes	yes	yes	yes	yes	yes	yes	yes	yes
		(b) Give reasons for non-participation at each stage	n.a.	n.a.	n.a.	n.a.	n.a.	n.a.	n.a.	yes	yes	n.a.
		(c) Consider use of a flow diagram	no	no	no	no	no	no	no	no	no	no
Descriptive data	14*	(a) Give characteristics of study participants (eg demographic, clinical, social) and information on exposures and potential confounders	yes	yes	yes	yes	yes	yes	yes	yes	yes	yes
		(b) Indicate number of participants with missing data for each variable of interest	yes	n.a.	n.a.	no	n.a.	n.a.	n.a.	n.a.	n.a.	no
		(c) *Cohort study* —Summarise follow-up time (eg, average and total amount)	n.a.	n.a.	yes	n.a.	n.a.	n.a.		n.a.	n.a.	
Outcome data	15*	*Cohort study* —Report numbers of outcome events or summary measures over time	yes	yes	yes	yes	yes	yes		yes	yes	
		Case-control study— Report numbers in each exposure category, or summary measures of exposure										
		Cross-sectional study— Report numbers of outcome events or summary measures							yes			yes

Main results	16	(*a*) Give unadjusted estimates and, if applicable, confounder-adjusted estimates and their precision (eg, 95% confidence interval). Make clear which confounders were adjusted for and why they were included	yes	yes	yes	no	yes	yes	yes	yes	yes	yes
		(*b*) Report category boundaries when continuous variables were categorized	n.a.	n.a.	yes	yes	yes	yes	n.a.	yes	yes	n.a.
		(*c*) If relevant, consider translating estimates of relative risk into absolute risk for a meaningful time period	n.a.	n.a.	n.a.	n.a.	n.a.	n.a.	n.a.	n.a.	n.a.	n.a.
Other analyses	17	Report other analyses done—eg analyses of subgroups and interactions, and sensitivity analyses	n.a.	yes	n.a.	yes	n.a.	n.a.	n.a.	yes	yes	yes
**Discussion**												
Key results	18	Summarise key results with reference to study objectives	yes	yes	yes	yes	yes	yes	yes	yes	yes	yes
Limitation s	19	Discuss limitations of the study, taking into account sources of potential bias or imprecision. Discuss both direction and magnitude of any potential bias	yes	yes	yes	yes	yes	yes	yes	yes	yes	yes
Interpretat ion	20	Give a cautious overall interpretation of results considering objectives, limitations, multiplicity of analyses, results from similar studies, and other relevant evidence	yes	yes	yes	yes	yes	yes	yes	yes	yes	yes
Generalisa bility	21	Discuss the generalisability (external validity) of the study results	yes	yes	yes	yes	yes	yes	yes	yes	no	yes
**Other information**												
Funding	22	Give the source of funding and the role of the funders for the present study and, if applicable, for the original study on which the present article is based	yes	yes	yes	no	yes	no	yes	yes	no	yes
